# Mechanical Support Strategies for High-Risk Procedures in the Invasive Cardiac Catheterization Laboratory: A State-of-the-Art Review

**DOI:** 10.3390/jcm12247755

**Published:** 2023-12-18

**Authors:** Niels T. A. Groeneveld, Carolien E. L. Swier, Jose Montero-Cabezas, Carlos V. Elzo Kraemer, Frederikus A. Klok, Floris S. van den Brink

**Affiliations:** 1 Department of Anesthesiology, Erasmus Medical Center, Dr. Molewaterplein 40, 3015 GD Rotterdam, The Netherlands; n.groeneveld@erasmusmc.nl; 2 Department of Intensive Care, Leiden University Medical Center, Albinusdreef 2, 2333 ZA Leiden, The Netherlands; c.e.l.swier@lumc.nl (C.E.L.S.); c.v.elzo_kraemer@lumc.nl (C.V.E.K.); 3 Department of Cardiology, Leiden University Medical Center, Albinusdreef 2, 2333 ZA Leiden, The Netherlands; j.m.montero_cabezas@lumc.nl; 4 Department of Medicine—Thrombosis and Hemostasis, Leiden University Medical Center, Albinusdreef 2, 2333 ZA Leiden, The Netherlands; f.a.klok@lumc.nl

**Keywords:** mechanical circulatory support, catheterization laboratory, cardiogenic shock

## Abstract

Thanks to advancements in percutaneous cardiac interventions, an expanding patient population now qualifies for treatment through percutaneous endovascular procedures. High-risk interventions far exceed coronary interventions and include transcatheter aortic valve replacement, endovascular management of acute pulmonary embolism and ventricular tachycardia ablation. Given the frequent impairment of ventricular function in these patients, frequently deteriorating during percutaneous interventions, it is hypothesized that mechanical ventricular support may improve periprocedural survival and subsequently patient outcome. In this narrative review, we aimed to provide the relevant evidence found for the clinical use of percutaneous mechanical circulatory support (pMCS). We searched the Pubmed database for articles related to pMCS and to pMCS and invasive cath lab procedures. The articles and their references were evaluated for relevance. We provide an overview of the clinically relevant evidence for intra-aortic balloon pump, Impella, TandemHeart and ECMO and their role as pMCS in high-risk percutaneous coronary intervention, transcatheter valvular procedures, ablations and high-risk pulmonary embolism. We found that the right choice of periprocedural pMCS could provide a solution for the hemodynamic challenges during these procedures. However, to enhance the understanding of the safety and effectiveness of pMCS devices in an often high-risk population, more randomized research is needed.

## 1. Introduction

Thanks to advancements in percutaneous cardiac interventions, an expanding patient population now qualifies for treatment through percutaneous endovascular procedures. Traditionally, mortality rates have been high among patients experiencing ischemia-related cardiogenic shock necessitating high-risk percutaneous interventions [1]. Nevertheless, eligibility for percutaneous interventions has extended far beyond coronary interventions. High-risk procedures, such as transcatheter aortic valve replacement, endovascular management of acute pulmonary embolism and ventricular tachycardia ablation, are increasingly being performed in the catheterization laboratory. Given the frequent impairment of ventricular (left or right) function in these patients, which can deteriorate during percutaneous interventions, it is hypothesized that mechanical ventricular support may improve periprocedural survival and subsequently patient outcome. Several techniques and devices have been introduced for ventricular support in patients undergoing high-risk procedures. However, several recent observational studies have questioned the safety and cost-effectiveness of hemodynamic support devices in patients undergoing percutaneous coronary intervention (PCI) [2,3]. Over the past two decades, substantial evidence has demonstrated that the routine use of an intra-aortic balloon pump (IABP) does not reduce 30-day mortality [3] or long-term mortality [4] in patients with acute ischemic cardiogenic shock undergoing early revascularization. Furthermore, the elective placement of an IABP during PCI also fails to decrease the risk of major complications [5]. Therefore, the routine use of an IABP is no longer recommended for the treatment of cardiogenic shock or during percutaneous interventions [6]. Since the introduction of the IABP, novel techniques have emerged. Among these innovations, percutaneous transvalvular microaxial flow pump devices like Impella and venoarterial extracorporeal membrane oxygenation (VA-ECMO) are demonstrating promising results. The aim of this review is to elucidate the most commonly used percutaneous mechanical circulatory support (pMCS) devices and strategies and to highlight future perspectives for mechanical support during high-risk procedures in the cardiac catheterization laboratory.

## 2. Intra-Aortic Balloon Pump

Since its introduction in 1961 [7], IABP has been commonly used for patients with cardiogenic shock. Through properly timed balloon inflation and deflation, occluding the aorta during diastole, IABP therapy augments coronary blood flow, facilitates left ventricular (LV) unloading and decreases afterload and myocardial oxygen consumption. Cardiac output increases by 0.5–1.0 L per minute [8]. The Seldinger technique is used for insertion through an 8 Fr arterial sheath, usually in the femoral or axillary artery, and the correct position (2–3 cm distally of the origin of the left subclavian artery) is ensured through angiography. Aortic regurgitation and dissection or aneurysm of the aorta are contraindications for insertion.

For decades, IABP has been the therapy of choice for cardiogenic shock and periprocedural support. However, due to the accumulating evidence mentioned before and the ESC Guideline 2016 recommendations [9], its use drastically decreased. Although primary insertion during high-risk procedures has declined, secondary insertion for total mechanical support (LV unloading during ECMO) or as a rescue therapy for prolonged postprocedural cardiogenic shock still occurs frequently. Also, due to its simplicity and wide availability, especially in smaller hospitals, utilization of IABP therapy has not been abandoned [10].

Complications of IABP therapy primarily result from insertion, malposition and prolonged use. Frequently observed complications include major bleeding (3.3%), peripheral ischemic complications (4.3%), sepsis (15.7%) and stroke (0.7%) [4]. Although rare, IABP misplacement could lead to major vascular complications (type A and B dissection and subclavian dissection) [11].

In addition to classic counterpulsation provided by IABP therapy, a novel device has been introduced in the last decade. The NuPulseCV intravascular ventricular assist system provides extended-duration ambulatory counterpulsation. It consists of a durable pump surgically implanted through the distal subclavian artery connected to subcutaneous electrocardiogram leads, providing the trigger source for the balloon [12]. An external driveline to a wearable drive unit provides compressed air for inflation and deflation of the balloon. A recent feasibility trial showed promising short-term outcomes in advanced heart failure patients, and larger trials are underway [13].

## 3. Impella (Abiomed, Danvers, MA, USA)

Impella provides cardiac mechanical support through an intravascularly inserted microaxial pump. It can either be used as a left-sided or right-sided ventricular support device. In the left-sided device (Impella 2.5, CP, 5, 5.5), the microaxial pump is placed beyond the aortic valve into the ventricle, directing blood from the left ventricle into the aorta. When dealing with right ventricular failure, a right-sided device can be utilized (Impella RP). In this device, the blood is pumped from the inferior vena cava to the pulmonary artery through a percutaneously inserted catheter. Both devices unload the ventricles, enhancing output and thereby improving coronary blood flow and end-organ perfusion. Hemodynamically, the Impella systems reduce myocardial oxygen consumption, improve mean arterial pressure and reduce pulmonary capillary wedge pressure [14,15]. In cases of biventricular failure, both devices can be employed simultaneously [16,17]. Depending on the type, a flow of 2.5 to 5.5 L per minute can be generated. Unlike the other left-sided systems, Impella 2.5 and Impella CP can be used percutaneously to provide short-term support (<4 days). Both the implantation and explantation of these devices are considered straightforward procedures [18], making this form of support suitable for use during high-risk percutaneous coronary intervention (HRPCI) and as rescue therapy in scenarios involving LV failure and cardiogenic shock during cath lab procedures. The remaining support devices require surgical placement but can support hemodynamics for up to 14 days or longer [19]. Unlike IABP therapy, Impella does not require wave triggering or ECG, facilitating stability even in the setting of tachyarrhythmias or electromechanical dissociation.

Several complications are associated with the use of Impella devices. These range from vascular complications, including limb ischemia (incidence 0.07–10%) and local insertion-related injuries (1.3–2%), to bleeding complications (0.05–54%) due to anticoagulation use, hemolysis and thrombocytopenia. Non-vascular complications, such as cardiac tissue damage arising from device migration (0.05–23%) or CVA (2.4–6.3%), may also occur, along with access site infection (1.1%) or sepsis (0.16–19%) [20]. Recent studies showed that Impella, when compared with IABP therapy, is associated with a greater risk of complications and significantly higher costs [2,21]. Consequently, a clear assessment must be made when employing these devices, especially during elective procedures.

## 4. TandemHeart (Cardiac Assist Inc, Pittsburgh, PA, USA)

The TandemHeart device facilitates LV volume unloading by withdrawing blood from the left atrium through a trans-septal 21 Fr cannula inserted in the femoral vein. The oxygenated blood is passed through a centrifugal flow pump and re-injected in the lower aorta or femoral artery through a 17–19 Fr cannula. It can provide up to 4 L per minute of circulatory support. Providing the cath team is trained for trans-septal insertion, the average implantation time is 45–60 min [22]. The TandemHeart device can also be configured to provide RV circulatory support. Through a dual-lumen cannula or two separated cannulas, blood is withdrawn from the right atrium or ventricle and into the pulmonary artery [23].

Although the TandemHeart device provides superior hemodynamic and metabolic support compared to IABP, no benefit in early survival has been observed in patients with cardiogenic shock [24]. Moreover, due to the highly invasive procedure, insertion leads to more serious adverse events. Therefore, TandemHeart is not recommended as the first-choice approach for cardiogenic shock [25].

## 5. VA-ECMO

The use of VA-ECMO in the cath lab is diverse. In the case of ST-elevation myocardial ischemia (STEMI) presenting with cardiogenic shock, VA-ECMO can be used to ensure end-organ perfusion. It is easily inserted percutaneously via the femoral artery and vein and can theoretically provide flows of up to 7 L per minute. In experienced hands, it can be rapidly inserted, buying time for further intervention. The optimal timing for VA-ECMO utilization in such a scenario remains controversial. When deployed prior to intervention, it can stabilize the patient and ensure adequate end-organ perfusion prior to PCI. It can ameliorate cardiogenic shock and can provide adequate support during (periprocedurally induced) electrical storm or cardiac arrest. However, one of the main drawbacks is that deployment of the VA-ECMO circuit can delay time to reperfusion, prolonging cardiac ischemia and increasing myocardial damage and infarct size. However, if VA-ECMO is deployed rapidly, the delay will be minimal with cannulation times as rapid as 10 min in experienced hands. In cardiac arrest, 10 min of deep hypoxia can be devastating for cerebral perfusion, and the preferred order of interventions should be carefully considered.

Besides the timing and sequence of intervention, complications of VA-ECMO with known significant associated morbidity must be considered as well. In general, the complications of VA-ECMO include major bleeding (incidence 26.8–56.6%), neurologic complications (9.9–17.7%), acute kidney injury (35.5–74%) and infections (19.5–44%) [26]. Specifically for peripheral VA-ECMO, one must be cautious of obstruction of the femoral artery, potentially leading to lower limb ischemia [27] (incidence 12.5–22.6%). Separate cannulation of the distal femoral artery with antegrade perfusion branching of the ECMO circuit is frequently used to prevent limb ischemia. Additionally, the competition between anterograde flow, driven by cardiac output, and retrograde flow generated by VA-ECMO [28] can lead to LV overload, pulmonary edema and ultimately the Harlequin syndrome. 

The use of VA-ECMO in out-of-hospital cardiac arrest is of special consideration as several recent trials on the subject have been published with conflicting results. The ARREST trial showed promising results in patients presenting with cardiac arrest [29], driven by a mean arrest-to-VA-ECMO time of 59 min and a survival of 43%. Consecutively, the PRAGUE-OHCA trial showed no difference in survival after OHCA in a per-protocol analysis; however, it showed a 9.5% difference in survival in favor of the ECMO group [30]. A post hoc analysis showed that if return of spontaneous circulation (ROSC) was obtained, survival was significantly higher in the ECMO group with an average arrest-to-ECMO time of 61 min. Third, the INCEPTION trial showed no difference in survival between the ECMO and the conventional group. However, arrest -o-VA-ECMO time was lengthy at 74 min [31]. Therefore, it was suggested that if the time from cardiac arrest to VA-ECMO was decreased, the chances of a favorable outcome would increase. The currently running ON-SCENE trial will hopefully answer the question of whether a short arrest-to-VA-ECMO time through pre-hospital ECMO insertion will increase survival. Potentially, this could mean that the role of VA-ECMO in the treatment of cardiac arrest will become more pre-hospital-based.

## 6. High-Risk Percutaneous Coronary Interventions

PCI may become a high-risk procedure [32] when dealing with patients with extensive comorbidities and/or reduced LV function (LVEF < 40%) combined with challenging coronary anatomies. These challenging coronary anatomies may involve multivessel disease, left main disease, extensive coronary calcification requiring debulking techniques and last remaining vessels. The performance of high-risk PCI can lead to a high rate of periprocedural complications and mortality [33,34]. pMCS is frequently used during PCI, aiming to prevent major adverse cardiac and cerebrovascular events (MACCEs). 

The PROTECT II study in 2012 compared Impella 2.5 support during high-risk PCI with support with an IABP [35]. It demonstrated that the 30-day incidence of MACCEs was not different for patients with IABP or Impella 2.5 support. However, trends for improved outcomes were observed for Impella 2.5-supported patients at 90 days. The subsequent study, the PROTECT III trial, compared the more powerful Impella CP to the historic Impella 2.5 cohort of the PROTECT II trial [36]. Here it demonstrated fewer MACCEs in the PROTECT III group at 90 days, and additionally, fewer bleeding complications were observed, and more complete revascularization was achieved. In addition to these results, Wollmuth et al. also showed significant improvement in the LVEF at 90 days in patients undergoing high-risk PCI with Impella support. Also, patients with more complete revascularization showed a greater increase in ejection fraction [37]. 

The timing of Impella in such a scenario, either pre- or postprocedure, appears to have no influence on clinical outcomes in the short or long term. Becher et al. showed no difference in the occurrence of MACCEs between patients with Impella 2.5 support before or after PCI [38]. However, the number of included patients was small, and it remains to be seen if larger future studies yield similar results. The meta-analysis by Leon et al. indicated that in patients experiencing acute myocardial infarction complicated by cardiogenic shock, initiating Impella preemptively before PCI resulted in improved 30-day survival compared to commencing Impella after PCI [39]. There was no significant difference between both groups in the occurrence of complications, as mentioned earlier in this article. The ongoing PROTECT IV trial aims to address Impella timing issues in patients undergoing high-risk PCI [40]. 

VA-ECMO can also be used for assistance in high-risk PCI [41]. Scarce data show promising results in performing high-risk PCI with the prophylactic use of VA-ECMO [42]. However, the sample size is small, and prophylactic VA-ECMO implantation prior to high-risk PCI is far from clinical practice in most hospitals. The now-recruiting CHIP-BCIS3 trial will, among other things, address this issue [43]. In comparison to the Impella device, VA-ECMO is relatively cheap. However, with the scarce evidence presently at hand, some consider VA-ECMO to be a more invasive strategy than Impella. 

A more novel technique, the PulseCath iVAC 2L, has been used in the recent past to provide pulsatile circulatory support during high-risk PCI. The documented sample size remains very small in the literature; however, it showed significantly more circulatory support compared to conventional IABP [44]. Comparing its performance against more frequently used continuous-flow devices in randomized controlled trials is needed to determine its applicability in clinical use.

As mentioned in the introduction, routine use of IABP is not recommended in patients with myocardial infarction and cardiogenic shock undergoing high-risk PCI, independently of the timing of insertion [45]. However, its role as a rescue therapy for subgroups remains unclear. Especially in patients with failed revascularization or persistent cardiogenic shock after PCI, evidence remains scarce. A small trial observed a non-significant but possibly clinically relevant advantage for IABP insertion in patients with post-PCI cardiogenic shock [46]. 

## 7. Ventricular Tachycardia Ablation

Ventricular tachycardia (VT), whether due to ischemic or non-ischemic cardiomyopathy, carries a high burden of morbidity and mortality. An implantable cardioverter–defibrillator (ICD) and anti-arrhythmic medications are the primary treatments, aimed at either terminating VT or reducing the risk of its occurrence. In cases of persistent VT with frequent ICD shocks or escalating anti-arrhythmic therapy, morbidity and mortality increase and quality of life decreases [47,48]. A catheter or radiofrequency ablation is an effective therapy recommended for a select group of patients according to the 2022 ESC guidelines for the management of patients with ventricular arrhythmias. This group includes patients with refractory symptoms of either ischemic or non-ischemic cardiomyopathy, which do not respond to or are intolerant of anti-arrhythmic medication [49].

In 11% of patients with scar-related VT, hemodynamic instability occurs during VT ablation due to the complexity of the substrate, concurrent heart failure, VT storm development, and the presence of comorbidities such as diabetes mellitus (DM) and chronic obstructive pulmonary disease (COPD). In this group, the success rate of the procedure is lower, and mortality is higher during follow-up [50]. To prevent hemodynamic instability during the procedure, pMCS may be considered.

Randomized controlled studies concerning the use of pMCS in VT ablations are still to be performed. Therefore, current recommendations are based on data from observational studies and meta-analyses.

In the meta-analysis by Turagam et al. from 2018, the effectiveness and safety of VT ablation with pMCS devices were examined. Among a total of 2026 patients, 284 received mechanical support during the procedure, mainly by Impella (2.5 and CP) and TandemHeart (percutaneous left atrial-to-femoral artery bypass using an external centrifugal pump). This pMCS group was generally sicker, with poorer LV function and more VT storms. No difference was found in the procedural success rate, VT recurrence and mortality at follow-up between the two groups. On average, the procedure took an additional 71 min in the pMCS group, and more complications were observed, including tamponade, bleeding, infarction and worsening of heart failure (RR 1.83, 95% CI (1.21–2.76), *p* = 0.004) [51].

The meta-analysis by Mariani et al. from 2021 showed similar results. They only described the prophylactic use of mechanical support during VT ablation. A total of 400 procedures were performed, with 187 involving pMCS devices. There were no baseline differences between both groups. Support was primarily provided with Impella 2.5 and CP (86.6%) and TandemHeart (13.4%). In the pMCS group, more VTs were induced, and patients remained longer in VT (24 min). The procedural success rate, 30-day mortality and the occurrence of complications were similar in both groups. Ultimately, 64% of patients without prophylactic support received mechanical support per procedure due to severe hemodynamic instability [52].

In both meta-analyses, VA-ECMO was rarely used and mainly used as a rescue therapy. The limited use of VA-ECMO as mechanical support during VT ablations is also reflected in the systematic review by Vallabhajosyula et al. from 2020, which examined short-term mortality (in-hospital or <30 days) with this form of support. A total of seven studies with 867 patients were included through a literature search over 20 years (2000–2019). Of these, 15% received support with VA-ECMO, mostly due to hemodynamic instability from VT storms. On average, 2–3 VTs were induced per procedure. Ablation times ranged from 34 min to 4.7 h, and the duration of ECMO support ranged from 140 min to 6 days. Short-term mortality in the pMCS group was 15% due to refractory VT, cardiac arrest and heart failure. The most common complications were bleeding/hemolysis (6.2%) and vascular injury (6.2%) [53].

## 8. Transcatheter Aortic Valve Replacement

Aortic valve stenosis is a prevalent valve disorder, associated with significant morbidity, mortality and societal disease burden. Its prevalence increases with age, rising from 0.2% in those aged 50–60 to 9.8% in those aged 80–89 years [54]. Aortic valve stenosis is a mechanical issue, and the definitive treatment involves either surgical aortic valve replacement (SAVR) or transcatheter aortic valve replacement (TAVR). Non-invasive treatment primarily focuses on symptom management and preventing disease deterioration but has a poor prognosis with a 5-year mortality of 50–60% and a 10-year mortality of 90% [55].

SAVR was once regarded as the gold standard for severe aortic valve stenosis. However, it cannot be performed in 32% of cases due to comorbidities or patient inoperability [56], and TAVR has been a viable alternative for both inoperable/high-risk patients and patients of age since its introduction in 2002 [57,58,59,60].

During the TAVR procedure, there are patient- and procedure-related factors that can lead to severe hemodynamic instability. Patient-related causes are mainly due to cardiopulmonary dysfunction and comorbidities typical of patients with severe aortic valve stenosis [61]. The cause of hemodynamic instability due to procedural factors should primarily be attributed to myocardial stunning, resulting from the valve placement itself, valve dislocation, severe regurgitation of the newly placed valve, coronary obstruction, annulus rupture, arrhythmias and/or tamponade [62]. Mechanical support during the procedure, either prophylactically or as a rescue strategy, can provide a solution.

As of now, there has been limited systematic research on pMCS during the TAVR procedure, making it challenging to draw definitive conclusions.

Through a systematic review, Vallabhajosyula et al. attempted to gain a better understanding of the indications, complications, and clinical outcomes of patients who underwent TAVR with periprocedural VA-ECMO support. A literature search from 2000 to 2018 identified nine studies involving a total of 5191 patients, examining short- and long-term mortality. In total, 3.9% of patients received support with VA-ECMO, primarily periprocedurally due to severe hemodynamic instability from procedure-related complications such as rhythm problems, annulus rupture, aortic valve regurgitation and main stem stenosis. Short-term mortality (in-hospital/30-day) was 29.8%, and 1-year mortality was 52.4%. Complications (bleeding, vascular injury, tamponade, cerebral ischemia and kidney failure) were noted in 10–50% of patients. Although this systematic review provides some insight into the type of patient and the effect of VA-ECMO use as a rescue therapy on mortality, it offers limited insight into the effects of prophylactic VA-ECMO during TAVR and the associated patient population [63].

The MUST registry attempted to address this by collaborating with 13 high-volume centers (>100 TAVRs per year) worldwide. Besides the use of VA-ECMO support, it also described other forms of pMCS. Its goal was to systematically collect data on mortality with pMCS use during the TAVR procedure in the short and long term (1 year). It also examined device-related complications. Over a 10-year period (2011–2020), 87 patients (76.5 ± 11.8 years, 63.2% male) were registered. These patients, with severe aortic valve stenosis, required mechanical support during TAVR (prophylactic 39.1%, rescue therapy 50.6%, postprocedure 10.3%) due to severe hemodynamic instability. In the majority, VA-ECMO was used (75.9%), followed by Impella CP (19.5%) and TandemHeart (4.6%). The preference for VA-ECMO is likely since VA-ECMO, unlike Impella and TandemHeart, does not interfere with the TAVR procedure and provides both cardiac and pulmonary support. In 10.3%, an IABP was placed with VA-ECMO for LV unloading. In-hospital and long-term mortality were 27.5% and 49.4%, respectively. Patients with prophylactically placed pMCS had lower periprocedure mortality than patients receiving pMCS as rescue therapy. There was no difference in survival between the different pMCS devices. Complications were observed in 57.3% of procedures (bleeding and vascular injury 21.8%, CVA 5.7% and myocardial infarction 8%), of which 10.3% were registered as device-related [62].

Specific research on Impella usage during TAVR encompasses a single-center study from 2020 by Amalla et al. In this prospective study, the authors looked at 30-day mortality with Impella (2.5 and CP) use during TAVR. Out of 390 patients, 10% received Impella support as rescue therapy for cardiogenic shock. The 30-day mortality was 40%. There were no cases of cerebral ischemia or vascular complications in this patient group [64].

## 9. Transcatheter Edge-to-Edge Repair Procedures

Mitral valve regurgitation (MR) is the most prevalent valve abnormality in the Western world, affecting approximately 1 to 2% of the population. MR is divided into primary and secondary mitral regurgitation (PMR/SMR). Its prevalence increases with age, with the valve abnormality occurring in almost 10% of the population over the age of 75. The left ventricle experiences dilatation due to volume overload, without a concurrent increase in muscle thickness (hypertrophy) [65]. Without adequate treatment, this cardiac remodeling can lead to heart failure and ultimately death [66].

For PMR, the 2021 ESC/EACTS guidelines for the management of valvular heart disease recommend surgical valve repair in patients with symptomatic severe mitral valve insufficiency (MR grade 3 or 4) and asymptomatic patients with poor LV function (LVEF ≤ 60% or LVESD ≥ 40%) or high pulmonary pressures (SPAP > 50 mmHg) and an estimated acceptable surgical risk. For patients with high surgical risk or patients who are deemed inoperable, transcatheter treatment is seen as a viable alternative, for which transcatheter edge-to-edge repair (TEER) is the most extensively studied and successful method [60,67,68,69,70]. With the introduction of the PASCAL transcatheter valve repair system, transcatheter options have further expanded, making priorly inoperable patients eligible for TEER [71].

Patients undergoing a TEER procedure can anticipate a moderate hemodynamic status due to valve insufficiency itself, cardiomyopathy, cardiac remodeling and the presence of comorbidities. Especially in the increasingly larger group of patients deemed unfit for surgery, pMCS can help maintain or optimize hemodynamics during the procedure, potentially leading to better patient outcomes. However, systematic research on this form of support during the TEER procedure has not been conducted to our knowledge. Evidence for the safety and effectiveness of these devices can currently only be based on information from a few case reports/series.

In these reports, pMCS was primarily employed as a rescue or bailout strategy for patients experiencing cardiogenic shock and severe MR, often secondary to various forms of cardiomyopathy. The most frequently utilized pMCS devices were the newer Impella (CP, 5 or 5.5) systems, known for their ability to generate high flows. In all cases, the use of the pMCS device, whether it was Impella, ECMO or a combination thereof, resulted in the technical success of the TEER procedure. Most of these patients could be weaned off pMCS devices and inotropes after the TEER procedure, with improvement in mitral valve insufficiency and heart failure symptoms. Only a few patients died due to multi-organ failure [72,73,74,75,76,77,78,79,80,81,82,83,84].

## 10. Catheter-Directed Therapies for Pulmonary Embolism

The global burden imposed by pulmonary embolism is high [85,86], not only due to immediate pulmonary embolism (PE)-related death, hospitalization and morbidity, but also after the initial diagnosis and treatment as up to half of the survivors describe a persistent decrease in functional capacity and quality of life [87], known as the post-pulmonary-embolism syndrome [88].

The clinical manifestations of acute PE within the first hours range from asymptomatic incidentally diagnosed PEs to obstructive shock requiring hemodynamic resuscitation and support, the latter referred to as high-risk PE. Treatment of high-risk PE is obviously directed to immediate reperfusion to relieve the right ventricle and to ensure systemic perfusion to prevent early PE-related death [89]. Catheter-guided therapies, sometimes combined with pMCS to preserve cardiac output, have been used to directly decrease the thrombotic burden. In non-high-risk patients, reperfusion therapy should be reserved for those in whom the anticoagulation treatment fails [90]. For decades, systemic thrombolysis has been the first-line reperfusion therapy in high-risk and non-high-risk acute PE [89,90]. 

However, the use of systemic thrombolytic therapy imposes a considerable 10–15% risk of major hemorrhage and a 2% risk of intracranial bleeding, leading to considerable mortality and morbidity. It is therefore discouraged in the event of absolute or relative contraindications, and only a small proportion of patients affected by high-risk PE ultimately receive systemic thrombolysis [91,92,93]. For these reasons, the mortality of high-risk PE remains high to date. Over the past 20 years, new catheter-directed reperfusion techniques have been developed, increasingly involving interventional cardiology and radiology in the management of acute PE, in high-risk as well as non-high-risk patients. Especially because of their considerably better safety profile over systemic thrombolysis, the use of catheter-guided therapies becomes more and more appealing in selected stable patients at high risk of decompensation and death. Notably, to date, besides case series and registries, no significant evidence is available for the use of catheter-directed therapies in PE patients. 

Catheter-directed therapies can grossly be divided into four main groups: thrombus fragmentation, aspiration embolectomy, rheolytic thrombectomy and ultrasound-accelerated catheter-directed thrombolysis, all showing promising results with low risks of bleeding [94,95,96,97,98,99]. It should be stated that for reperfusion therapy to be successful (hemodynamic improvement), complete clot removal is not necessary. Clear criteria for treatment success are not available. Even so, immediate improvement of hemodynamic (heart rate, PAP, cardiac output) and respiratory vital parameters are strong markers of adequate treatment response and can be used to stop the catheter treatment. It should also be emphasized that anticoagulation during and after completion of the catheter-guided procedure remains the keystone of successful PE treatment. 

For patients with PE who are hemodynamically stable, the question remains which patients need more aggressive therapy to prevent morbidity and mortality. Further stratification and choice of definitive therapy of this subgroup through RV function assessment [89,100,101,102] and troponin levels [103,104,105] is preferably guided by a PERT, consisting of all locally available specialties that are involved in the treatment of severe PE, e.g., a vascular or pulmonary physician, a cardiac surgeon, an interventional cardiologist and/or an intensive/critical care specialist. A post hoc analysis of the PEITHO trial suggested that adding clinical characteristics of patients on top of biomarkers and evaluation of RV function allows for better identification of stable patients who may benefit from upfront reperfusion [106]. Several large randomized controlled trials comparing catheter-directed therapy to the standard of care in hemodynamically stable patients are being performed to provide evidence and guide PERT decisions [107,108,109,110,111].

When focusing on catheter-guided therapy, pMCS can be used as prophylactic periprocedural support or as a bail-out for periprocedural refractory hemodynamic instability. Due to the fact that there are no primary indications at the moment for catheter-guided therapy in patients with massive or submassive PE, most literature describes pMCS as a rescue therapy in patients with severe hemodynamic instability. The nature of shock (obstructive right ventricular failure) at least requires right ventricular support. 

A right-sided Impella device (Impella RP) can provide hemodynamic support in patients with refractory shock due to PE. Impella RP showed significant hemodynamic improvement in post-cardiotomy syndrome or in acute myocardial ischemia [16]. For PE, however scarce, several case reports show survival benefit using Impella RP devices with high rates of successful weaning of the support device and discharge from the hospital [112,113].

Patients who received mechanical support via VA-ECMO in these case reports also had favorable outcomes regarding device weaning and hospital discharge. However, when compared to Impella RP devices, the numbers were lower [114]. A small case cohort showed 95% survival at 90 days when VA-ECMO was used as hemodynamic support in high-risk PE patients with refractory shock and end-organ failure [115]. However, a recent review showed no overall short-term benefit and only suggested its use in patients < 60 years or post-surgical embolectomy [116]. Bleeding complications and availability are remaining concerns. 

For other pMCS devices, only small case series are available, and recommendations are limited. Four patients with massive PE were treated with a percutaneous RVAD device. All patients showed RV recovery [117,118]. 

To date, evidence on the timing and type of pMCS in hemodynamically stable and unstable PE patients is scarce, and future research is needed to guide optimal treatment [89,100,101,102,103,104,105,106].

## 11. Summary, Conclusions and Future Perspective

As a result of an aging population and advancements in healthcare, we are increasingly encountering patients with extensive comorbidities and end-stage cardiovascular failure that require medical treatment [119]. In high-risk patients, such as patients with a need for acute coronary revascularization, acute reperfusion due to PE, persistent rhythm problems despite adequate drug therapy, and valve abnormalities such as severe aortic valve stenosis and mitral valve insufficiency, transcatheter treatment serves as a viable alternative to surgical or solo drug therapy [59,60,120]. Despite the minimally invasive nature of procedures in the catheterization room, a higher likelihood of periprocedural complications and mortality has been found when these procedures are performed in this high-risk population [33,34,50,61]. Theoretically, periprocedural pMCS in the form of an Impella or ECMO device could provide a solution during these procedures, by maintaining and optimizing coronary and organ perfusion with the aim of improving future clinical outcomes for the patient. An overview of the most frequently used pMCS is provided in Figures S1 and S2 [23]. Based on the literature found, although still scarce for many procedures in the cath lab, this theory appears to apply. 

Studies on the prophylactic use of ECMO during high-risk PCIs show promising results [42]. This contrasts with prophylactic Impella use, where there is still much uncertainty about the optimal timing. The ongoing PROTECT IV trial aims to address this issue [40]. This multicenter randomized controlled trial builds upon previous PROTECT trials and investigates the effect of preventive Impella support on MACCEs in patients undergoing high-risk PCI.

Although pMCS support during the TAVR procedure is rarely necessary, positive results on clinical outcomes also seem to apply to it. It can be used not only as prophylactic support during high-risk procedures, but also as a rescue strategy in cases of hemodynamic failure. Moreover, the expected complication risk related to pMCS devices seems relatively low [62,63,64].

Less favorable results seem to exist for pMCS support during VT ablations. Even though hemodynamics stabilizes during the procedure with pMCS support, allowing for more extensive and prolonged treatment, this improvement in procedural aspects does not translate into better outcomes. Both procedural success and mortality do not improve with pMCS use compared to those for patients who do not receive mechanical support [51,52]. Impella appears to be the preferred device during VT ablation. This preference is likely because ECMO support can only be applied in specialized centers; it is a more invasive procedure, and there is a higher risk of thromboembolism, bleeding and stasis in the left ventricle, which complicates VT mapping and ablation [121].

Little is known about the effects of pMCS devices on clinical outcomes during the TEER procedure. Our knowledge is completely based on case reports/series, so it is hard to draw any conclusions about this topic. 

For patients with refractory shock and PE, catheter-directed therapies show promising results, especially regarding the safety of the procedure. pMCS devices (right-sided mechanical support and VA ECMO) are more readily available and could provide partial or full hemodynamic support in these patients. However, robust data on short- and long-term patient outcomes are lacking, as are data on the recommended catheter system and the use of pMCS devices during catheter-directed therapy. Preferably, in the absence of contraindications, high-risk PE should therefore be treated with full systemic thrombolysis and intermediate-risk PE with anticoagulation alone, while awaiting new data. The use of catheter-directed therapy and pMCS devices should depend on availability and local expertise.

Besides the use of pMCS in a specific procedure, much can be learned from the use of pMCS in patients with cardiogenic shock. In general, randomized controlled trials comparing ECMO with less-invasive pMCS are needed to support the recent increase in ECMO use. A similar gap in evidence applies to the use of total mechanical support combining ECMO with Impella or IABP to facilitate increasingly complex procedures or support in cardiogenic shock. Also, because of its widespread use due to its low costs and simplicity, evidence and recommendations for IABP therapy are needed to specify its exact indications besides the negative recommendation for its use in high-risk PCI.

For most procedures, the use of pMCS is predominantly either preemptive or as a bail-out strategy. Since its use is almost exclusively related to high-risk patients, baseline mortality and adverse events are high. Consecutively, limited data are available, and guidelines generally suggest the use of pMCS as a last-resort therapy. Future recommendations on the deployment of pMCS as a standard of care should be based on randomized evidence from upcoming trials. Until then, a multidisciplinary approach should be sought to guide pMCS decisions for these high-risk and complex patient populations.

The need for novel techniques is never-ending, and improving minimally invasive techniques could potentially aid a large population in need of cardiovascular intervention. To enhance the understanding of the safety and effectiveness of pMCS devices in this often high-risk population undergoing a minimally invasive procedure in the cath lab, further systematic and randomized research is required.

## Data Availability

Not applicable.

## Supplementary Materials

The following supporting information can be downloaded at: https://www.mdpi.com/article/10.3390/jcm12247755/s1, Figure S1: Left ventricular circulatory support devices. Figure S2: Right ventricular circulatory support devices. Table S1: Percutaneous mechanical circulatory support.
