# Supplementary material for: Mechanical Support Strategies for High-Risk Procedures in the Invasive Cardiac Catheterization Laboratory: A State-of-the-Art Review
Source: J Clin Med. 2023 Dec 18;12(24):7755. doi: 10.3390/jcm12247755 (PMC10744085; doi:10.3390/jcm12247755)
Supplement: Supplementary file 1 [file jcm-12-07755-s001.zip › jcm-2729996-supplementary.pdf]

Journal of
Clinical Medicine

MDPI

Supplementary Materials

Figure S1: Left ventricular circulatory support devices

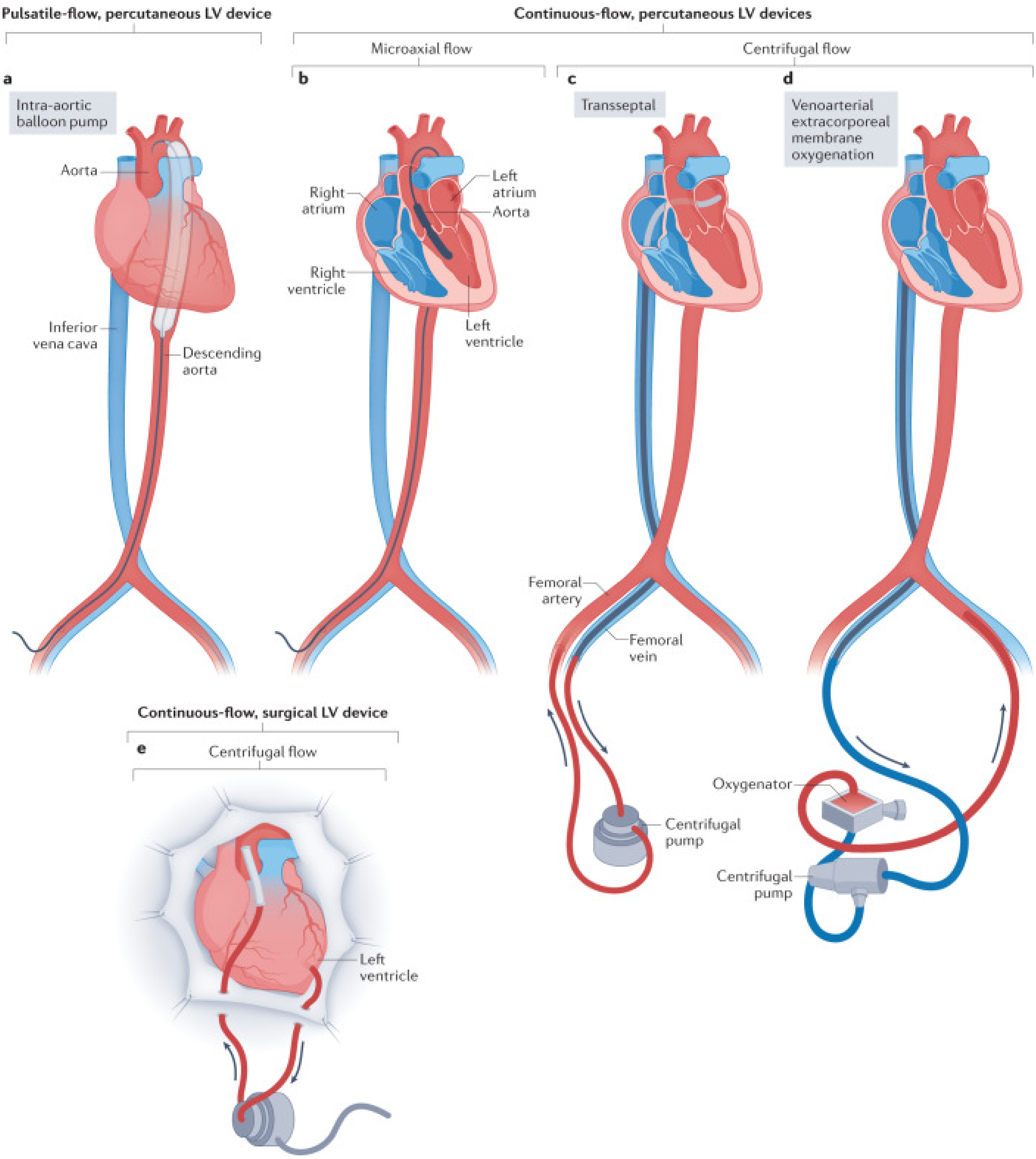


**a**, The intra-aortic balloon pump uses counterpulsation to provide circulatory support and is commonly inserted via the femoral or axillary artery. **b**, Microaxial flow devices traverse the aortic valve via the femoral or axillary artery and

continuously remove blood from the left ventricular (LV) cavity to achieve unloading. **c**, Transseptal percutaneous assist devices are powered by a centrifugal pump and are inserted via the femoral vein, across the intra-atrial septum and into the left atrium, with an outflow cannula in the femoral artery. Devices using centrifugal pumps require a separate control and monitoring module. **d**, Venoarterial extracorporeal membrane oxygenation uses a centrifugal pump to pull venous blood from the right atrium, through an oxygenator and into the arterial circulation via the outflow cannula in the aorta. Common cannulation sites include the femoral or axillary vessels or via central cannulation by thoracotomy or sternotomy. Extracorporeal membrane oxygenation can be used for left, right or biventricular support. **e**, Surgical extracorporeal centrifugal devices provide LV support via sternotomy or minimally invasive thoracotomy. Inflow cannulation is from the left ventricle or left atrium, and outflow cannulation is into the aorta. Image courtesy of Salter et al, Nat Rev Cardiol. 2023 Apr; 20(4):263-277 (23).

Figure S2: Right ventricular circulatory support devices

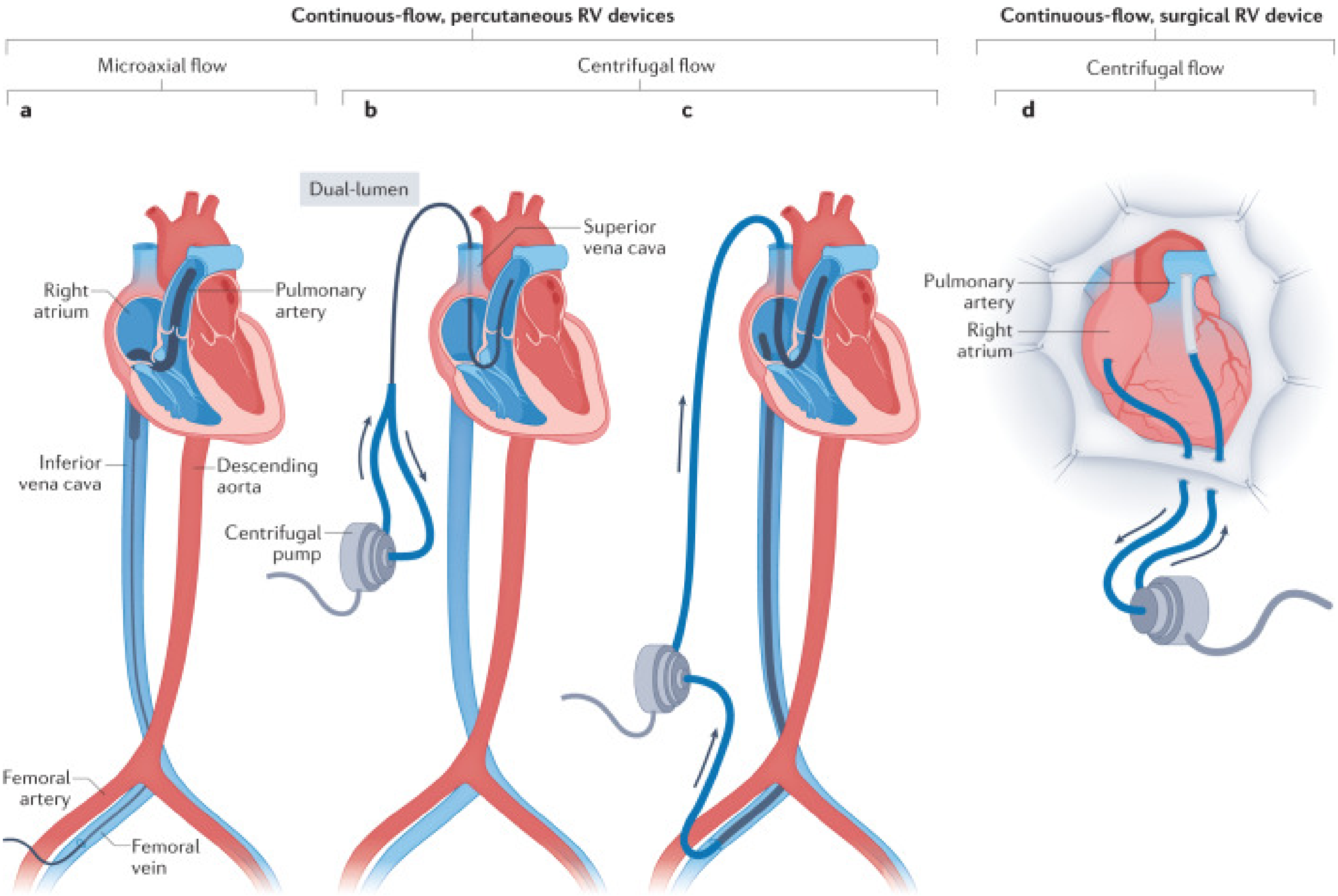


**a**, Microaxial flow devices for right ventricular (RV) support are inserted percutaneously through the femoral vein and into the pulmonary artery. **b**, Dual-lumen percutaneous assist devices draw blood from the right atrium or right ventricle to an external continuous centrifugal pump and out to the pulmonary artery. **c**, Percutaneous cannulas draw blood from the right atrium to a continuous centrifugal pump and into the pulmonary artery via the femoral, internal jugular or subclavian vein. **d**, Surgical extracorporeal centrifugal devices provide RV support via sternotomy or minimally invasive thoracotomy. Inflow cannulation is from the right atrium

or right ventricle and outflow cannulation is into the pulmonary artery. Image courtesy of Salter et al, Nat Rev Cardiol. 2023 Apr; 20(4):263-277(23).

Table S1: Percutaneous Mechanical Circulatory Support

| | Pump mechanism | Catheter/Cannula size | Benefits | Contraindications | Complica-tions | Considerations |
|---|---|---|---|---|---|---|
| IABP | Pneumatic | 8 Fr | Low cost Simplicity | Aortic regurgitation, aortic dissection or aneurysm, uncontrolled sepsis, severe peripheral artery disease (relative) | Bleeding, limb ischemia, stroke, sepsis | Combined use with VA-ECMO for LV unloading |
| Impella 5.0 | Axial Flow | 22 Fr | Minimal invasive LV support, no ECG triggering required | LV thrombus, severe aortic stenosis (relative), severe peripheral artery disease (relative) | Perforation, vascular injuries, bleeding, arrythmia, aortic regurgitation | High costs Combined use with VA-ECMO for LV unloading |
| Impella RP | Axial Flow | 22 Fr | Minimal invasive right ventricular support, no ECG triggering required | Pulmonary artery abnormalities, pulmonary or tricuspid regurgitation or mechanical valves, mural thrombus of right atrium or vena cava | Perforation, vascular injuries, bleeding, arrythmia, pulmonary valve regurgitation | High costs Minimal invasive support for patients with high-risk PE |
| TandemHeart | Centrifugal flow | 21 Fr inflow, 15-17 Fr outflow | Superior hemodynamic support | RV failure, ventricular septal defect, severe aortic regurgitation, severe | Major vascular injury, limb ischemia, bleeding | Invasive procedure Anticoagulation |

| | | | | | | |
|---|---|---|---|---|---|---|
| | | | | peripheral artery disease (relative) | | |
| VA-ECMO | Centrifugal flow | Femoral: 21-27 Fr inflow, 15-23 Fr outflow | Superior hemodynamic support | Pre-existing condition incompatible with recovery, end-stage malignancy, severe aortic regurgitation, uncontrolled bleeding, severe peripheral artery disease (relative) | Major vascular injury, limb ischemia, bleeding | Invasive procedure Anticoagulation |
